# Learning visual to auditory sensory substitution reveals flexibility in image to sound mapping

**DOI:** 10.1038/s41539-025-00385-4

**Published:** 2025-12-03

**Authors:** Asa Kucinkas, Chrysa Retsa, Peter B. L. Meijer, Mark T. Wallace, Monica Gori, Micah M. Murray

**Affiliations:** 1 The Radiology Department, Lausanne University Hospital and University of Lausanne, Lausanne, Switzerland; 2 The Sense Innovation and Research Center, Lausanne and Sion, Lausanne, Switzerland; 3 Metamodal BV, Eindhoven, The Netherlands; 4 Department of Psychology, Vanderbilt Brain Institute, Vanderbilt University, Nashville, TN USA; 5 The Unit for Visually Impaired People, Italian Institute of Technology, Genoa, Italy

**Keywords:** Neuroscience, Psychology, Psychology

## Abstract

Visual-to-auditory sensory substitution devices (SSDs) translate images to sounds. One SSD, The vOICe, translates a pixel’s vertical position into pitch and horizontal position into time. This mapping is primarily based on technical considerations for preserving image content in human-audible sounds without presupposing intuitiveness, although some literature also invokes crossmodal correspondences in perception, such as pitch for elevation. We investigated these presuppositions and the efficacy of learning a traditional algorithm (i.e., pitch indicating elevation and time indicating azimuth) versus a reversed algorithm (i.e., pitch indicating azimuth and time indicating elevation), or an arbitrary single-tone control mapping (i.e., each visual stimulus was represented by a single non-systematic pitch–time pairing without structured spatial correspondences). Sixty sighted adults participated with random assignment to the Traditional, Reversed, or Control groups. They completed learning and evaluation sessions using simplified black-and-white visual stimuli. Both the Traditional and Reversed groups learned mappings within 30 minutes and demonstrated successful recognition of novel stimuli, outperforming the Control group but not differing between them. Structured mappings facilitate SSD learning. Mapping pixel position onto spectral-temporal acoustic axes appears flexible, rather than anchored to cross-modal correspondences. These findings reveal how SSDs may be rendered bespoke across user, stimuli, and functionality levels.

## Introduction

Sensory substitution refers to the process by which information normally received through one sensory modality is instead conveyed through another, using a device that translates stimuli from the original sense into an alternative sensory modality^1^. The concept of sensory substitution dates back several decades, trying to move beyond early innovations such as the white cane and Braille serving as foundational tools for individuals with visual impairments. Over time, more sophisticated systems have been developed to provide alternative sensory inputs to compensate for the loss of vision. Among these innovations is The vOICe^2^, pioneered by Peter Meijer, which translates visual information into auditory stimuli. In the version used in this study, The vOICe converts a 64 × 64-pixel image into a mono “soundscape”, where frequency corresponds to the vertical position, time represents the horizontal position, and amplitude conveys pixel brightness.

Numerous studies have demonstrated the efficacy of The vOICe in enabling blind and visually-impaired individuals to recognize objects and navigate environments^3–5^. This auditory information is processed by cross-modal brain circuits, recruiting regions typically involved in high-level visual functions—such as face or letter recognition—to interpret soundscapes, thereby opening possibilities for functional vision rehabilitation^6–8^. Recruitment may also involve low-level visual areas, as evidenced by studies showing that rTMS over occipital cortex (including V1) disrupts the ability to interpret soundscapes^9^, and that fMRI showing reveals increased V1 activation in trained blind users of The vOICe^10^. These early works primarily emphasized the efficacy of The vOICe for object recognition and navigation. Others studies have examined how performance is affected either by specific settings of the image-to-sound conversion or by altering the conversion parameters themselves. For instance, Brown et al. (2011)^11^ systematically varied sonification settings (e.g., dark-to-loud vs. bright-to-loud) and found that “loud object vs. silent background” performed best for recognition. Stiles & Shimojo (2015)^12^ demonstrated that auditory sensory substitution can feel intuitive for naïve users under certain mappings, but that a modified encoding such as scanning top-to-bottom with high pitch on the right (i.e., a 90° axis swap) significantly impairs interpretation. Wright & Ward (2013)^13^ extended this line of inquiry by applying interactive genetic algorithms to optimize vOICe-like parameters, suggesting that algorithmic fine-tuning can enhance efficiency and user experience in SSDs. These studies indicate that the underlying algorithmic functionality plays a critical role in shaping learning, intuitiveness, and overall performance with SSDs.

Cross-modal correspondences—systematic associations between features of different sensory modalities—may play an important role in sensory substitution design. These correspondences are thought to be grounded in both innate neural mechanisms and experience-based associations^14^. For instance, higher-pitched sounds are commonly associated with brighter and higher-positioned visual stimuli^15–18^; a relationship that aligns with The vOICe’s traditional mapping (though see also^19,20^ for examples of more nuanced relationships in visually-impaired and blind individuals). Such natural mappings may enhance intuitiveness and learning efficiency in sensory substitution, as they align with pre-existing perceptual biases^21^. Consequently, the traditional algorithm of The vOICe, which adheres to these purported correspondences, may offer advantages over alternative mappings (though there is also some weaker evidence for pitch-laterality associations, especially in musicians^18,22^). The effectiveness of sensory substitution devices (SSDs) is heavily dependent on the design and optimization of their underlying algorithms, which determine how visual inputs are mapped to auditory (or tactile) outputs. Fine-tuning these spectral-temporal mappings could enhance the clarity, efficiency, and usability of SSDs, making them more intuitive and bespoke for the individual. Yet despite these prior explorations of vOICe parameter space, there is still limited empirical work directly comparing fundamentally different axis mappings (e.g., pitch-as-elevation vs. pitch-as-azimuth), leaving open the question of whether cross-modal correspondences constrain learning.

**Fig. 1:**
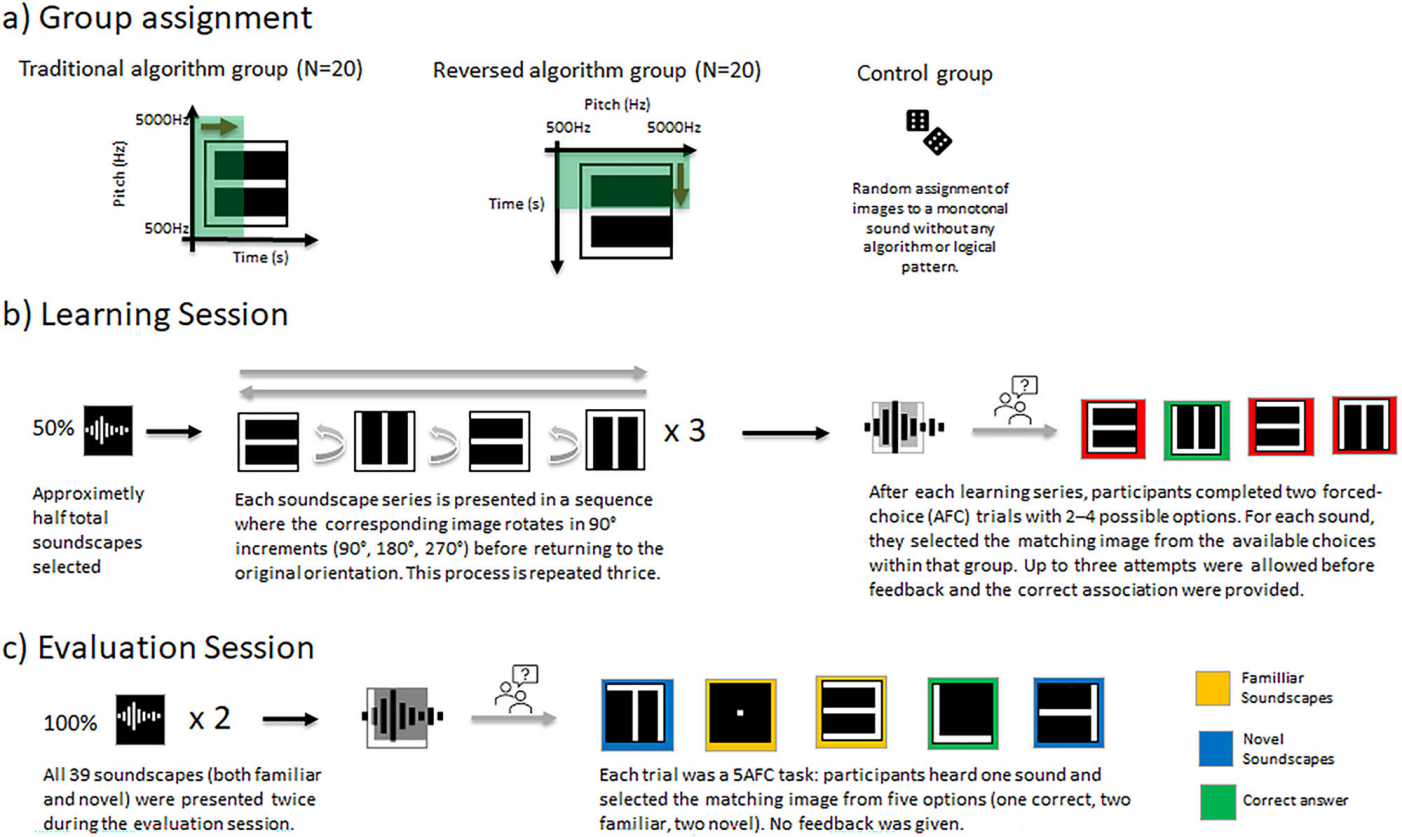
SSD algorithms and study design. The figure provides a schematic illustration of the algorithms used for each group and of the study design. **a** Participants (N = 60) were assigned to one of three groups according to the conversion algorithm they experienced. Then, they each completed a learning phase (**b**) immediately followed by an evaluation phase (**c**).

**Fig. 2:**
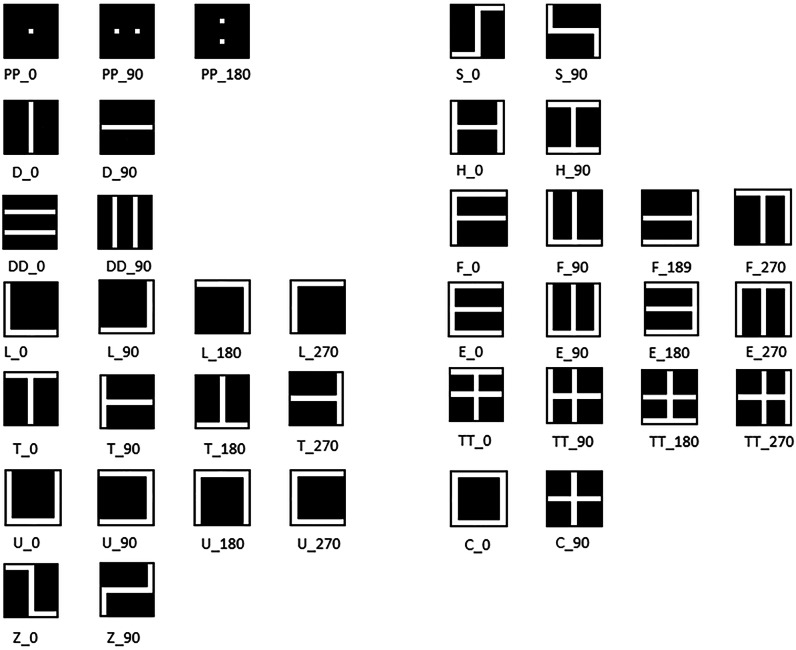
Stimulus set. The figure displays the full set of all 39 stimuli presented during the experiment.

This study addressed the performance of The vOICe versus an alternative mapping. In the traditional algorithm, low-to-high pitch encodes elevation from bottom to top, while left-to-right scanning over time encodes horizontal position. In the reversed algorithm, low-to-high pitch instead encodes horizontal position from left to right, while scanning over time encodes elevation from top to bottom (as illustrated in Fig. 1). By way of example, a horizontal line would be converted by the traditional algorithm as a narrow-band sound persisting for a relatively long duration. The same horizontal line would be converted into a broadband sound persisting for a relatively short duration by the reversed algorithm. For the control group, each visual stimulus was paired with an arbitrary unique single tone lacking spatial correspondences. During the learning session, participants were exposed to half of the stimulus set. During the evaluation session, the full stimulus set was presented (Fig. 2). If both algorithms yield similar performance, this suggests that learning processes can override potential pre-existing sensory mappings or that these mappings are not determinative of performance, indicating that adaptability plays a more significant role than cross-modal associations.

## Results

### Learning Phase

A one-way between-subjects ANOVA was conducted to examine the effect of mapping condition (Traditional, Reversed, Control) on percentage accuracy during Attempt 1. Descriptive data (mean ± SEM) revealed clear differences between groups. The Traditional group achieved 90.00 ± 1.85%, the Reversed group 84.17 ± 2.65%, and the Control group 72.08 ± 3.02% accuracy. The analysis showed a significant main effect of Condition (F_(2, 57)_ = 12.81, *p* < 0.001, ${\eta }_{{\rm{P}}}^{2}=0.310{\omega }^{2}=.282$), indicating that mapping condition significantly influenced performance on the first attempt. Post hoc comparisons using the Sidak correction revealed that the Traditional group performed significantly better than the Control group (p < 0.001) and that the Reversed group also performed significantly better than the Control group (p = 0.004). There was no significant difference between the Traditional and Reversed groups (p = 0.299). The Levene’s test for homogeneity of variances was not significant (F_(2, 57)_ = 2.014, p = 0.143), confirming that the assumption of equal variances was met. Only Attempt 1 was analysed in this model to avoid potential bias, as not all participants completed the subsequent attempts with all stimuli.

### Evaluation Phase

To assess the effect of group differences on task performance, a 3 × 2 mixed-model repeated measures ANOVA was conducted on both accuracy scores and processing time. Processing time (PT) in this study is defined as the duration, measured in seconds, from the moment participants were presented with the five response options in the forced-choice task until they selected an answer. The analysis included three groups (Traditional, Reversed, and Control) and two stimulus types (Familiar and Novel), where Group was treated as the between-subjects factor, and Stimulus Type as the within-subjects factor.

### Accuracy Scores

**Fig. 3:**
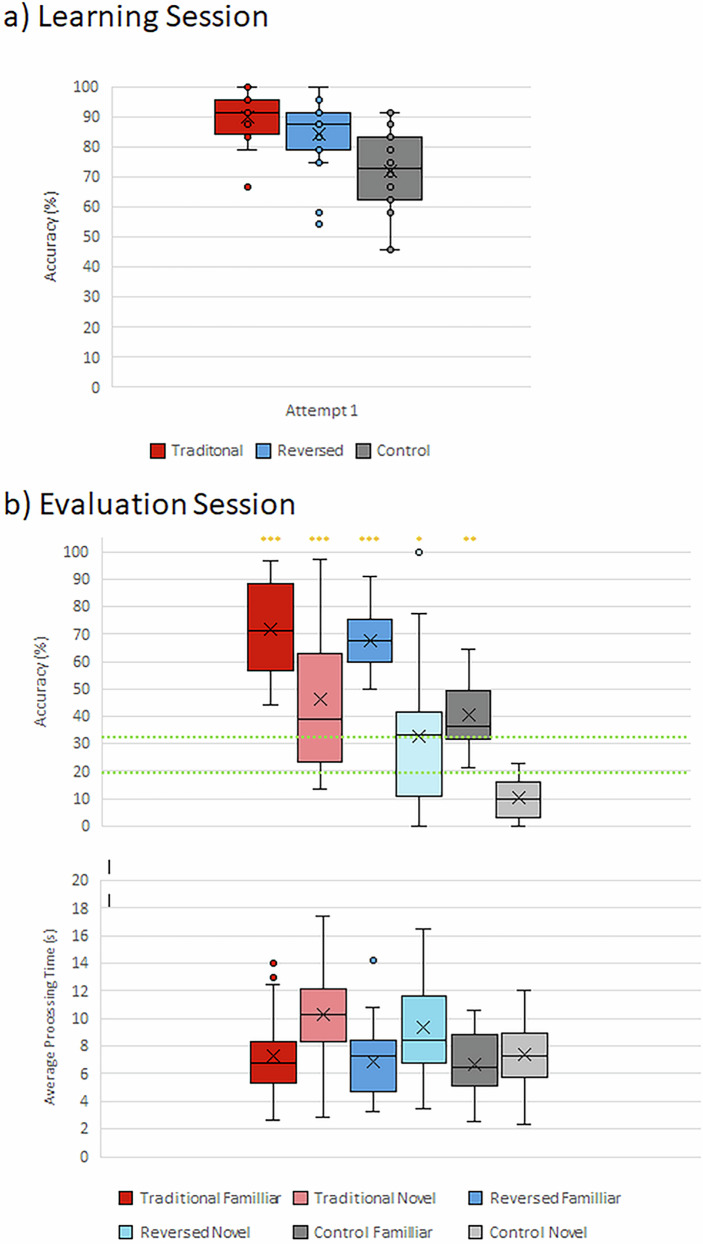
Performance metrics. **a** Accuracy rates during the learning session are plotted as a function of Group (Traditional, Reversed, Control) for the first attempt only. **b** Performance during the evaluation session are plotted as a function of Group (Traditional, Reversed, Control) as well as whether the stimuli were familiar or novel. The upper panel plots accuracy (chance levels for the 5-AFC = 20% and a more conservation 33% for the possible strategy of first discriminating novel vs. familiar before item-specific recognition, are indicated by the green dashed lines and p-values versus 20% chance are indicated by the gold asterisks; *≤0.05; **≤0.01; ***≤0.005). The lower panel plots processing time. In both panels, the centre line displays the mean, the “x” displays the median, the box displays the 25–75% range, the whiskers 1.5 times the interquartile range, and circles (“o”) display outliers.

Accuracy by stimulus type and group is presented in Fig. 3b. A mixed-model repeated measures ANOVA revealed a significant main effect of Group on accuracy (*F*_(2, 57)_ = 27.95, *p* < 0.001, ${{{\eta }}^{2}}_{{\rm{p}}}=0.495$). Participants in both the Traditional and Reversed groups outperformed those in the Control group. Post-hoc comparisons (2-sided unpaired t-tests) using Bonferroni correction indicated that the Traditional group showed significantly higher accuracy than the Control group (*p* < 0.001, M₍diff₎= 33.63%), as did the Reversed group compared to the Control group (*p* < 0.001, M₍diff₎= 24.79%). However, the difference between the Traditional and Reversed groups was not statistically significant (*p* = 0.189, M₍diff₎= 8.84%). There was also a significant main effect of Stimulus Type (*F*_(1,57)_ = 135.03, *p* < 0.001, ${{{\eta }}^{2}}_{{\rm{p}}}=0.703$) Participants achieved higher accuracy for familiar stimuli (*M* = 60.29%, *SD* = 19.31) than for novel stimuli (*M* = 29.83%, *SD* = 25.78). The interaction between group and stimulus type was not significant (*F*_(2,57)_ = 0.883, *p* = 0.419, ${{{\eta }}^{2}}_{{\rm{p}}}=0.030$) indicating that the accuracy difference between familiar and novel stimuli was consistent across groups. To assess whether participants performed above chance, one-sample t-tests were conducted for each group and stimulus type. Results revealed that accuracy for familiar stimuli was significantly above chance in all three groups: Traditional (*t*_(19)_ = 14.73, *p* < 0.001), Reversed (*t*_(19)_ = 19.37, *p* < 0.001), and Control (*t*_(19)_ = 7.25, *p* < 0.001). For novel stimuli, accuracy was significantly above chance in the Traditional group (*t*_(19)_ = 4.61, *p* < 0.001) and the Reversed group (*t*_(19)_ = 2.19, *p* = 0.041), but not in the Control group (*t*_(19)_ = -5.65, *p* < 0.001).

### Processing Time

Processing times for each stimulus type and group are presented in Fig. 3c. The Traditional group exhibited the longest processing times for novel stimuli, whereas the Control group had the shortest overall processing times. A significant main effect of *Stimulus Type* was found, *F*_(1,57)_ = 98.337; *p* < 0.001; ${{{\eta }}^{2}}_{{\rm{p}}}=0.633$, indicating that participants took significantly longer to process novel stimuli compared to familiar stimuli. Additionally, a significant interaction between *Stimulus Type* and *Group* was observed (*F*_(2,57)_ = 10.697, *p* < 0.001, ${{{\eta }}^{2}}_{{\rm{p}}}=0.273$ suggesting that the extent to which processing time increased for novel stimuli varied by group.

Post-hoc Sidak-adjusted comparisons (2-sided, unpaired t-tests) revealed that the Traditional group exhibited significantly longer processing times for novel compared to familiar stimuli (*p* < 0.001). The Reversed group showed a similar pattern (*p* < 0.001), and the Control group also demonstrated a significant increase in processing time for novel stimuli (*p* < 0.001), although the magnitude of this increase was smaller compared to the other two groups.

The main effect of *Group* was not significant (*F*_(2,57)_ = 1.999, *p* = 0.145, ${{{\eta }}^{2}}_{{\rm{p}}}=0.066$), indicating that group membership alone was not a strong predictor of overall processing time differences. These findings suggest that differences in processing time were primarily driven by whether the stimuli were familiar or novel, rather than by group membership alone.

### Debrief Questionnaire

Participant responses to the debrief questionnaire were analysed across the three experimental groups: Traditional, Reversed, and Control. Notable differences emerged in the use of strategies, perceived visualizability of the soundscapes, and confidence during the task. A high proportion of participants in both the Traditional (95%) and Reversed (89.5%) groups reported employing specific strategies during the learning phase, compared to 85.7% in the Control group. Participants in the Traditional (65%) and Reversed (78.9%) conditions also more frequently reported that they would be able to draw the soundscapes they heard, relative to only 28.6% of the Control group.

When reflecting on the learning phase, 90.0% of Traditional participants and 89.5% of Reversed participants stated they were confident when giving correct responses, compared to 85.7% in the Control group. Confidence on incorrect responses was lower overall but varied by group: 40% of Traditional participants felt confident when incorrect, whereas 31.6% of Reversed and 57.1% of Control participants expressed confidence despite providing incorrect answers.

Regarding the evaluation phase, 55.0% of Traditional participants and 68.4% of Reversed participants reported being aware when they made errors, while only 47.6% of the Control group indicated such awareness. Confidence patterns during evaluation differed across groups. The Reversed group displayed the highest overall confidence, with 27.3% of participants feeling confident “often,” 33.3% “half the time,” and 26.7% “very rarely.” The Control group followed a similar trend, though confidence was more polarized, with 27.3% reporting “often,” 27.8% “half the time,” 40.0% “very rarely,” and 5.0% “never.” In contrast, the Traditional group showed the lowest confidence overall, with only 15.0% reporting “often,” 35.0% “half the time,” and half of participants (50.0%) “very rarely.”

A one-way ANOVA was performed to test for group differences on the key measures. Results indicated a significant group effect for the ability to draw soundscapes (*F*_(2,57)_ = 6.51, *p* = 0.003, ${{{\eta }}^{2}}_{{\rm{p}}}=0.186$). Post-hoc Sidak comparisons revealed that both the Traditional (p = 0.041) and Reversed (p = 0.003) groups reported significantly higher visualizability than the Control group, whereas the difference between the Traditional and Reversed groups was not significant (p = 0.721). No significant differences were found for strategy use (*F*_(2,57)_ = 0.48, *p* = 0.622, ${{{\eta }}^{2}}_{{\rm{p}}}=0.016$), confidence on incorrect answers (*F*_(2,57)_ = 0.96, *p* = 0.387, ${{{\eta }}^{2}}_{{\rm{p}}}=0.033$), confidence on correct answers (*F*_(2,57)_ = 0.10, *p* = 0.902, ${{{\eta }}^{2}}_{{\rm{p}}}=0.004$), or awareness of errors during evaluation (*F*_(2,57)_ = 0.88, *p* = 0.421, ${{{\eta }}^{2}}_{{\rm{p}}}=0.030$).

Participants described a diverse set of cognitive strategies for interpreting the sound–image associations. The most prevalent approach overall was pitch-based mapping, reported by 6 participants in the Traditional group, 5 in the Reversed group, and 8 in the Control group (19 in total). This strategy involved linking high- and low-pitched sounds to spatial dimensions (e.g., high sounds corresponding to upper or rightward image positions).

The second most common approach was mental imagery, mentioned by 3 Traditional, 6 Reversed, and 3 Control participants (12 in total). These participants described *imagining or visualizing* the soundscapes in their minds—mentally reconstructing image shapes or trajectories based on auditory cues.

Motor or gestural strategies (e.g., tracing shapes with the hand or head) were less frequent, appearing in 1 Traditional, 1 Reversed, and 2 Control participants (4 total). Temporal strategies relying on sound duration or rhythm were identified in 2 Traditional and 1 Reversed participants (3 total), while spatial orientation cues (e.g., left/right or top/bottom positioning) appeared only once in the Traditional group. Associative memory strategies—linking new sounds to remembered cues—were mentioned by a single participant in the Reversed condition. Finally, other reasoning approaches, such as attending to sound texture or imperfections, were reported by 5 Traditional, 3 Reversed, and 5 Control participants (13 total). Overall, participants combined multiple perceptual and mnemonic strategies, with pitch-based mapping and mental imagery emerging as the dominant approaches.

## Discussion

The present study examined different algorithmic implementations on the sensory substitution device, The vOICe. The findings demonstrate a successful and rapid learning process, with participants achieving above-chance performance in less than 30 minutes, both with a traditional algorithm representing a pixel’s vertical position by pitch and its horizontal position by time and also with a reversed algorithm representing a pixel’s vertical position by time and its horizontal position by pitch. With both algorithms, participants were able to accurately and efficiently recognize soundscapes following the learning session. This continued into the evaluation session, with proficiency demonstrated by the two algorithmic groups (Traditional and Reversed) significantly outperforming the Control group. This result provides a clear indication of efficient learning of a sensory substitution device (SSD) in a short time period that extended across both familiar and novel stimuli (at least with the stimulus set used here). As such, they open the possibility for future work to determine whether a given algorithm is better suited to specific individuals, as well as whether the discrimination or recognition of certain stimulus types might benefit more from presentation and learning with one algorithm versus another. Furthermore, the rapid learning process observed in our participants—achieving above-chance performance within a single 30-minute session—opens new avenues for investigating different aspects of The vOICe system, including the neurobiological mechanisms underlying perceptions induced via sensory substitution.

Notably, the speed of learning observed in this study is substantially faster than that reported in several previous studies using sensory substitution. For example, Arno et al.^23^ required ten one-hour learning sessions for participants to achieve proficiency in recognizing visual patterns using via an auditory substitution system. Their results reflected gradual improvement over multiple sessions, highlighting the need for prolonged exposure and repeated practice to generalize learning to novel patterns. However, it is important to note that Arno et al. used a pixel-to-frequency mapping system based on a simplified retina model, whereas The vOICe employs a spectral-temporal mapping approach, which may facilitate a different learning process. While using The vOICe, Kim and Zatorre^5^ trained participants on significantly more complex stimuli over 18 hours of instruction spread across three weeks. Other studies with other SSD methods have successfully used short training sessions for both navigation^24,25^, localization^26,27^, and object recognition^28–30^ tasks. Given these variations in stimulus complexity, learning protocol, and encoding mechanisms, direct comparisons are not straightforward.

Nonetheless, the markedly shorter learning time observed in our study may be attributed to the use of simplified stimuli and stimuli restricted to cardinal orientations, which likely reduced task difficulty. Moreover, our learning protocol incorporated cognitively supportive elements, including immediate feedback, structured forced-choice tasks, repeated exposure, and an explicit focus on mental imagery. These features are known to enhance perceptual learning efficiency and implicit pattern acquisition^31–34^, and recent works are implementing such strategies for learning SSDs^35,36^. Together, they may have facilitated the rapid internalization of auditory–visual mappings. This discrepancy in learning speed highlights the importance of task design and suggests that algorithmic learning can be accelerated through strategically crafted learning paradigms, opening the door to tailored sensory substitution protocols that adapt to individual users’ needs and abilities. This will be particularly important in the application of learning/training regimes for visually-impaired and blind users of this any other SSDs^25,29,37–39^.

The results of the learning accuracy analysis for Attempt 1 revealed a significant main effect of mapping condition, indicating that the type of algorithmic structure had a substantial influence on initial learning performance. Participants in both the Traditional and Reversed mapping groups achieved significantly higher accuracy than those in the Control condition, while no significant difference was found between the two algorithmic mappings. This pattern suggests that the presence of a structured sound–image translation algorithm—regardless of its specific axis orientation—facilitated learning performance, whereas the absence of such structure markedly hindered accuracy. The large effect size ($\eta$² =0.31) further underscores the strength of this relationship, with approximately one-third of the variance in performance explained by mapping condition. To avoid potential bias arising from the task design—where participants who answered correctly on earlier attempts did not proceed to subsequent attempts—only Attempt 1 was analysed, providing a conservative but robust estimate of early learning differences between groups. These findings reinforce the interpretation that structured, algorithm-based mappings substantially enhance the acquisition of novel auditory–visual correspondences, even during the very first exposure, and warrant future investigation with larger samples and more complex or less familiar stimuli to probe the limits of this early learning advantage.

Furthermore, this study provides some challenges to the assumption that the traditional algorithm is particularly intuitive and thus easier to learn or apply. Participants did not exhibit significant differences in performance between the traditional and reversed algorithms. The results call into question the assumption that the natural association of high-frequency sounds with higher spatial positions (as employed in the traditional The vOICe algorithm) is universally the most effective approach for image-to-sound conversion, though there is some evidence for the influence of crossmodal correspondences on localization^12,26^ and colour discrimination^40^ tasks using SSDs. That said, it is important to acknowledge that we did not capitalize upon the stereo panning feature of the current version of The vOICe, wherein a pixel’s horizontal position is translated into spatial audio cues. Nonetheless, our simplified algorithms yield similar behaviour, evidently yielding equivalent functional outcomes. This finding is particularly interesting in relation the notion of to cross-modal correspondence—the phenomenon where certain sensory stimuli are naturally associated with others, such as high-pitched sounds being linked to higher spatial positions or brighter colours^21,41^. It should be noted, however that there is evidence for other cross-modal correspondences that could potentially be exploited in the continued design of SSDs^42^.

Spence and Deroy have demonstrated that while cross-modal correspondences are widespread, they can be overridden by learning. In particular, statistical correspondences—such as pitch–size associations shaped by environmental exposure—are especially susceptible to modification^21^. While cross-modal correspondences have been reported in pre-verbal infants, the contribution of statistical regularities in environmental exposure cannot be excluded as a contributing factor. Our findings support the view that cross-modal correspondences are malleable and that learning can attenuate intuitive cross-modal mappings and potentially reduce their influence on perception (though see also^40,43^ for evidence of reversion to presumed cross-modal correspondences despite training with an alternative association). Although no significant behavioural differences were observed between the traditional and reversed algorithms, future research should examine the underlying neurophysiological processes elicited by each. At the neural level, the algorithms may function differently than what is apparent behaviourally, potentially due to differences in mental imagery, acoustic mapping, perceptual analysis strategy, or cognitive load. Research has shown that sensory substitution engages a complex interplay of brain regions traditionally associated with vision, audition, and multisensory processing^44,45^. However, it remains unclear whether such activation—particularly in the visual cortex—reflects visual processing or mental imagery^6,9^. Striem-Amit et al.^46^ provide evidence that visual cortex recruitment in blind individuals occurs predominantly after successful training with sensory substitution devices, suggesting that activation may be linked to learned, higher-order representations rather than automatic visual processing^46^. In the context of sighted individuals, this may imply a differential reliance on visual cortex activation depending on training depth or individual strategies. Graulty et al.^47^ found that even brief exposure to an SSD based on the Meijer algorithm elicited early (150–210 milliseconds) and late (420–480 milliseconds) ERP modulations post-training, localized to temporal and occipital regions. These findings support the notion of rapid, algorithm-specific cross-modal integration, although the precise contribution of visual versus imagery-based processes remains an open question^48^.

Additionally, research has further shown that auditory stimuli can automatically activate the visual cortex, improving subsequent visual discrimination, even when the auditory cue is non-predictive or task-irrelevant^49–51^. This suggests that different acoustic mappings may recruit distinct cross-modal networks, particularly within the superior temporal cortex, which integrates auditory and spatial information, and the occipital cortex, which adapts through sensory substitution^52^. Studies on auditory scene analysis (ASA) suggest that enhancing perceptual segmentation in frequency-based mappings, such as those used in The vOICe, can improve the extraction of structural patterns from sonified images^53^, though perhaps at the expense of effective frame rate for real-time use. The effectiveness of different mappings likely depends on how efficiently users can learn to separate and interpret auditory components, which could engage distinct cognitive and perceptual strategies. As such, to the extent that the traditional and reversed algorithms use different segmentation cues (e.g., pitch-based vs. time-based distinctions) to extract the same information about the referent image, they may impose differential cognitive demands, even if their final behavioural performance appears similar.

Cognitive load may also play a crucial role in modulating learning efficiency, particularly during its early stages as investigated here. According to Cognitive Load Theory^31^, learning is optimized when cognitive load is minimized (though see also ref. ^54^) for evidence that ~85% performance levels are optimal for learning), allowing for more efficient schema acquisition. If one algorithm aligns more closely with natural cross-modal correspondences—such as the association between pitch height and spatial elevation^41^ --it may reduce extraneous cognitive effort, leading to faster implicit learning^21^. Processing time could serve as a valuable metric for evaluating learning efficiency, as studies have shown that reaction times decrease for familiar stimuli due to automatization and implicit learning^34^. This principle may also extend to novel stimuli, with a more effective learning algorithm leading to shorter processing times and more efficient implicit learning. However, in the present study, no significant main effect of Group was observed for processing time, indicating that the overall speed at which participants responded did not differ substantially across the Traditional, Reversed, and Control conditions. This suggests that group membership alone was not a strong predictor of baseline processing efficiency.

Nonetheless, a significant main effect of stimulus type on processing time indicates that novel stimuli require significantly more time to process across all groups. Furthermore, an interaction between stimulus type and group was observed, reflecting that the increase in processing time for novel compared to familiar stimuli was not uniform across algorithms. Specifically, the Reversed group showed the largest increase in processing time for novel stimuli ( + 3.05 seconds), followed by the Traditional group ( + 2.53 seconds). In contrast, the Control group exhibited a smaller, though still statistically significant, increase ( + 0.83 seconds). This pattern suggests that participants in the algorithmic conditions engaged in more deliberate and cognitively demanding processing when confronted with novel stimuli, possibly due to the activation of structured mappings they had internalized during training. The smaller increase in the Control group may indicate a shallower or less systematic processing strategy, likely stemming from the absence of a consistent auditory-to-visual mapping. This suggests that even randomized auditory cues may contain residual structure—recurring patterns or statistical regularities that participants were able to detect—which aids in processing. However, this structure is far less effective than the explicit algorithm-based mappings, which offer a more systematic correspondence between auditory and visual elements. Thus, while all groups took longer to process novel stimuli, those trained with structured algorithms appeared to engage in more effortful, perhaps more meaningful generalization — a finding that warrants further investigation into the cognitive mechanisms underlying these differences. Future research with a larger sample size and longer and/or longitudinal training times (allowing for a transition to subconscious “automatic” processing of at least simple shapes) may help identify more nuanced effects, particularly in detecting subtle differences in learning efficiency between algorithmic mappings.

The debrief responses suggest that participants in the Traditional and Reversed conditions not only engaged more actively with the task but also developed more coherent internal representations of the auditory stimuli. The higher use of strategies and the greater ability to visualize soundscapes in these groups point toward deeper sensory integration and learning. Notably, this subjective sense of visualizability differed significantly between groups, with participants in the algorithm conditions more likely to report that they could draw the soundscapes they heard (*p* = 0.003), suggesting a more intuitive grasp of the sound–space relationship

Although participants in the algorithm groups reported using strategies more frequently overall, the types of strategies they employed were largely comparable to those observed in the Control group. Across all groups, pitch-based mapping and mental imagery emerged as dominant strategies (used by 19 and 12 participants respectively). Interestingly, the Control participants—despite lacking an explicit mapping rule—showed similar descriptive patterns, suggesting that they intuitively applied comparable logic to link sound and spatial features. This pattern indicates that the task itself may naturally elicit such strategies. Overall, the convergence across groups suggests that the ability to form internal associations between sound and space can arise spontaneously, even without explicit training.

Although differences were not statistically significant, participants in the algorithm groups tended to show greater metacognitive accuracy—being more confident when correct and less so when wrong—suggesting more reliable internal feedback. In contrast, the Control group exhibited more frequent misjudgements, indicated by lower confidence on correct answers and higher confidence when wrong, as well as limited ability to recognize errors during evaluation. This pattern may reflect a lack of consistent mapping or feedback, which impeded implicit learning. Overall, these findings reinforce the notion that the algorithm groups achieved comparable perceptual integration and subjective insight, supporting their potential advantage for early-stage sensory substitution learning.

One limitation of this study lies in the control condition setup. The control stimuli consisted of single-tone sounds, randomly assigned to images without any structured logic. However, single-tone sounds differ significantly from the structured audio cues used in the test algorithms, making differentiation inherently more difficult. An alternative would have been to use the same structured sounds as in the experimental conditions but assign them to images in a randomized manner. This adjustment would allow for stronger comparisons between groups and lead to more insightful conclusions regarding the impact of algorithm design on sensory substitution learning. A potential downside of this alternative, however, could be that such soundscapes would not be “neutral” but rather would run counter to any intuitions about potential rulesets or cross-modal correspondences.

Relatedly, the use of a 5-alternative forced choice task makes it conceivable that participants are first assessing if the soundscape is familiar vs. novel in order to reduce the alternatives. This would yield a floor performance level of 33% rather than 20%; both of which are indicated in Fig. 3B. While this would presumably apply to all groups, it may apply deferentially to the Traditional and Reversed groups and thus impact the sensitivity of the current experiment when contrasting with the Control group.

While we included a debrief questionnaire, it would undoubtedly be informative to explicitly ask participants what they understood of the image-to-sound conversion algorithm and to even draw the referent images/objects of a given soundscape.

Another limitation is that the study only measured short-term learning effects. Future studies should assess long-term retention and generalization of learned mappings, as well as whether users develop automaticity with more extended practice. On a similar line, this study only used very basic black and white soundscapes. It would be interesting to apply this learning method to more complex stimuli in the future to investigate other features of the converted images, such as their localization or physical complexity.

Another important question is the generalizability of these findings to blind individuals, as our results are based on sighted participants. For non-congenitally blind participants, cross-modal correspondences could serve as an important foundation for learning the algorithm. However, it is also possible that these correspondences would be similar or even less significant than for sighted individuals, given their differing sensory experiences and neural adaptations^55,56^.

This study examined the comparative effectiveness of two spectral-temporal translation algorithms in The vOICe sensory substitution device. We found that performance on both learning and evaluation sessions were indistinguishable across groups exposed to soundscapes based on a traditional algorithm (i.e. low-to-high pitch indicating elevation from bottom to top, and time indicating azimuth from left to right) or a reversed algorithm (i.e. low-to-high pitch indicating azimuth from left to right, and time indicating elevation from top to bottom). On the one hand, these findings open new possibilities in how SSDs may be rendered bespoke to individual users, specific categories of stimuli, or functionalities (e.g. object recognition, reading, or navigation). On the other hand, by showing that different algorithms are equally effective, these findings indicate flexibility in mapping between visual and auditory features (at least for a given task). This mapping is both rapidly achieved and does not appear strongly anchored to specific notions of pitch-elevation cross-modal correspondences.

## Methods

### Participants

Sixty sighted, adult participants with normal hearing (29 males, 31 females; age range: 18–27 years, *M* = 23.34) volunteered to participate and provided written informed consent to procedures approved by the cantonal ethics committee (CER-VD protocol #2018-00240) and conforming to principles outlaid in the 2013 Declaration of Helsinki.They were randomly assigned to one of three groups: the Control Group, the Traditional Algorithm Group, or the Reversed Algorithm Group (Fig. 1).

Before beginning the experiment, all participants in the Traditional Algorithm and Reversed Algorithm groups were informed that the sounds and images they would encounter were systematically related. They received a written explanation of the algorithm stating:

“*In this experiment, you will listen to soundscapes and view corresponding images. Each sound corresponds to a specific image following a logical system. The translation of each image into a sound is based on two dimensions: the DURATION and PITCH of the sound*.”

No additional details regarding the algorithm’s functionality were provided. Importantly, the control group did not receive the sentence about the translation being based on two dimensions, as this was not applicable to their condition. Instead, they were shown:

*“In this experiment, you will listen to soundscapes and view corresponding images. Each sound corresponds to a specific image.”*

Following these explanations, all participants received an identical briefing on the structure of the experiment, including its division into two phases and the procedures involved. Additionally, each sound sequence began with a distinct “click” sound, to which participants had the opportunity to listen twice before starting the task. They were also reminded to actively visualize the sounds after hearing them and before seeing the corresponding image (though we did not screen for aphantasia among our participants). Before the experiment, participants completed a brief questionnaire to assess potential confounding factors, such as hearing ability, musical background, dominant hand, and native language. One participant was excluded due to a technical issue during the experiment; however, no participants were excluded based on the predefined exclusion criteria, such as hearing impairment or uncorrectable vision deficits.

### Stimuli

The task employed was adapted from Arno et al^23^, who used various simple letters and shapes, such as the letter ‘E, with each stimulus having multiple variants created by rotating it in 45° increments. In the case of ‘E, this resulted in eight different variations. In our adaptation, we retained only the 0°, 90°, 180°, and 270° orientations. The rationale behind excluding 45°-angled images was to focus on the effect of the different algorithms, rather than to demonstrate the functionality of The vOICe system for more complex images, which has already been demonstrated by previous studies (e.g., ref. ^8^). Therefore, 39 different simple stimuli were utilized for this study (Fig. 2). The soundscapes generated from these stimuli were created using The vOICe^2^ and more specifically the open source code version available here: https://www.seeingwithsound.com/im2sound.htm#artificial_scenes. Only black and white images were used, eliminating the different amplitude aspects of the algorithm, in order to focus on frequency-time mappings without additional amplitude-based variables. Before each soundscape, a brief onset ‘click’ was presented at t = 0 for 3.3 ms, serving as a cue for the start of the 1.05 s sonification that came directly after. In the version used in this study, The vOICe converts a 64 × 64-pixel image into a mono “soundscape”, where frequency corresponds to the vertical position, time represents the horizontal position, and amplitude conveys pixel brightness. More recent versions of The vOICe pixelate the image into a 176 × 64 matrix and also add stereo panning for conversion of horizontal position of a pixel.

For the Traditional Algorithm group, the algorithm was applied such that the y-axis of 64 rows represented frequencies ranging from 500 to 5000 Hz, with higher rows corresponding to higher frequencies. This frequency mapping follows a logarithmic scale, this approach aligns with the human auditory system’s perception of pitch, which is also logarithmic in nature, ensuring a more natural auditory representation of visual information. The x-axis of 64 columns represented time, with each column corresponding to approximately 16.4 milliseconds, resulting in a total soundscape duration of 1.05 seconds, creating a 64×64-pixel soundscape matrix. For the Reversed Algorithm group, time was represented on an inverted y-axis, moving from the top of the matrix to the bottom, while frequency was on the x-axis, increasing from left to right. The control group experienced a single-toned constant sound corresponding arbitrarily to an image, with no underlying algorithm as illustrated in Fig. 1. The frequency of each sound was randomly selected within a range of 500 Hz to 5000 Hz, with each image assigned a unique frequency that differed by at least 50 Hz from its nearest neighbouring sound.

### Learning Session

Participants were seated in a quiet room, facing a computer screen positioned at eye level, which displayed the stimuli. To ensure consistency across all participants, the screen’s brightness, image size (64×64 pixels) with a screen resolution of (2560 × 1664), viewing distance (60 cm), and audio volume were standardized. Participants listened to the sounds through the computer’s built-in speakers.

The task began with a learning phase, where participants in each group underwent a sequence as follows: they initially heard a soundscape specific to their group’s algorithm and, two seconds later, were presented with the corresponding image. Participants were instructed to mentally visualize the image before viewing it. This sequence continued with the same image being rotated in 90° intervals (i.e. 90°, 180°, and 270°). Once the series completed its rotations, it was played in reverse order, starting from the final rotation and reverting back to the original image. Some images did not have four different angles. In these cases, the series was shorter. This entire learning sequence was repeated three times for each soundscape. There were also a few seconds pause between each rotation of the soundscapes.

After a sequence had been completed, participants listened to two of the soundscapes they had just learned and then answered two separate forced-choice questions, each paired with a unique soundscape selected from the set they had just learned. For each question, all previously learned visual options (between 1 and 4) were displayed as possible answers. They had up to three attempts to get the correct answer, once all attempts had been completed, they subsequently received feedback on the correct answer, meaning they heard the correct soundscape answer associated with the correct image.

The order of soundscapes presented was randomized, except for the first two sequences: the simple dot, the two-dot images (PP0, PP90, PP180), and the line image (D0, D90) which were shown first in order the facilitate learning as they are the most basic soundscapes. The six soundscape groups used in both the learning and evaluation phases were randomly selected and presented in a random order for each participant. This means that the specific groups trained and subsequently tested were not fixed across participants, and any six out of the twelve available groups could be included. The only exception to this full randomization concerned the first two sequences mentioned above. After these introductory sequences, all remaining soundscape groups were drawn and ordered randomly, ensuring that each participant encountered a distinct subset and sequence of soundscapes during the learning phase, and correspondingly, during evaluation.

Only about half of the total soundscapes were included in the learning phase, depending on which groups of soundscapes were randomly selected. For example, if the ‘E’ group was selected, it contained four variations (E0, E90, E180 and E270), whereas the ‘H’ group included only two (H0 and H90). This explains the approximate ‘half’—the exact number of soundscapes varied depending on the specific groups chosen for each participant. Regardless, all participants were taught a total of six soundscape groups out of the twelve available, as shown in Fig. 1. The remaining soundscapes were reserved for the evaluation phase. The learning phase took less than 30 minutes, and all groups of soundscapes selected were gone through twice. This learning session was adapted from the Kim and Zatorre^5^ study, in which participants were successfully trained to accurately recognize complex soundscapes with varying position, orientation, and size. Of note, participants were encouraged to form mental images of the soundscapes before visualization to aid learning. This instruction was provided in text format at the beginning of the learning session.

### Evaluation Session

In this phase, which took place immediately following the Learning session, participants were presented with either a novel soundscape (i.e. one not included in the learning session) or a familiar soundscape from the learning session. Their task was to select the correct image from five options, which included the correct answer, images corresponding to two novel soundscapes, and images corresponding to two previously learned soundscapes. While this design aimed to balance the representation of familiar and novel options, it could theoretically allow participants to reduce their uncertainty by inferring whether the correct answer was likely to belong to the “larger” category (familiar or novel). For example, if more familiar than novel options were shown, participants might intuit that the correct answer was among the familiar ones, effectively increasing the chance level from 20% to approximately 33%. However, this effect would apply equally across all conditions and is therefore unlikely to introduce systematic bias between groups, though it may slightly reduce the overall sensitivity of the task. Participants were evaluated on all the soundscapes learned during the learning session, along with approximately 20 novel soundscapes—selected based on the random allocation in the learning session—that were reserved for evaluation. Each soundscape in the evaluation session was presented twice in a fully randomized order. Unlike the learning session, participants did not receive feedback on their performance during this evaluation session.

### Debrief Questionnaire

The debrief questionnaire was administered at the end of the study while participants were still unaware of their individual performance outcomes. It combined closed- and open-ended items designed to capture participants’ subjective experiences and strategies throughout the experiment. Binary (Yes/No) questions assessed whether they had employed specific strategies during the task, believed they could visually reproduce the soundscapes, or had general awareness of making incorrect responses during the evaluation phase. When participants answered “Yes” to the strategies question, they were invited to briefly describe the approach or reasoning they had used.

To assess confidence, participants were asked to reflect on their overall experience rather than on individual trials or stimuli. They were asked to do so separately for the learning and evaluation phases. Multiple-choice questions prompted them to indicate whether, in the learning phase when they answered incorrectly, they had been confident in their responses or merely guessing, and vice versa for when they had answered correctly. A similar question was asked for the evaluation phase. Additionally, a Likert-type item (administered only for the evaluation phase) assessed how often they felt confident in their correct answers (“Always,” “Often,” “Half of the time,” “Very rarely,” “Never”).

## Data Availability

The data that support the findings of this study are available from the corresponding author upon reasonable request.

## Code availability

Scripts for this experiment can be found at https://github.com/line-neurophysiology-lab/SSD-The-vOICe.
